# Evaluating spatially enabled machine learning approaches to depth to bedrock mapping, Alberta, Canada

**DOI:** 10.1371/journal.pone.0296881

**Published:** 2024-03-27

**Authors:** Steven M. Pawley, Lisa Atkinson, Daniel J. Utting, Gregory M. D. Hartman, Nigel Atkinson

**Affiliations:** Alberta Geological Survey, Alberta Energy Regulator, Edmonton, Alberta, Canada; Bristol-Myers Squibb Company, UNITED STATES

## Abstract

Maps showing the thickness of sediments above the bedrock (depth to bedrock, or DTB) are important for many geoscience studies and are necessary for many hydrogeological, engineering, mining, and forestry applications. However, it can be difficult to accurately estimate DTB in areas with varied topography, like lowland and mountainous terrain, because traditional methods of predicting bedrock elevation often underestimate or overestimate the elevation in rugged or incised terrain. Here, we describe a machine learning spatial prediction approach that uses information from traditional digital elevation model derived estimates of terrain morphometry and satellite imagery, augmented with spatial feature engineering techniques to predict DTB across Alberta, Canada. First, compiled measurements of DTB from borehole lithologs were used to train a natural language model to predict bedrock depth across all available lithologs, significantly increasing the dataset size. The combined data were then used for DTB modelling employing several algorithms (XGBoost, Random forests, and Cubist) and spatial feature engineering techniques, using a combination of geographic coordinates, proximity measures, neighbouring points, and spatially lagged DTB estimates. Finally, the results were contrasted with DTB predictions based on modelled relationships with the auxiliary variables, as well as conventional spatial interpolations using inverse-distance weighting and ordinary kriging methods. The results show that the use of spatially lagged variables to incorporate information from the spatial structure of the training data significantly improves predictive performance compared to using auxiliary predictors and/or geographic coordinates alone. Furthermore, unlike some of the other tested methods such as using neighbouring point locations directly as features, spatially lagged variables did not generate spurious spatial artifacts in the predicted raster maps. The proposed method is demonstrated to produce reliable results in several distinct physiographic sub-regions with contrasting terrain types, as well as at the provincial scale, indicating its broad suitability for DTB mapping in general.

## Introduction

The thickness of surficial materials covering the bedrock surface (depth to bedrock, or DTB) represents an important consideration in earth and environmental sciences. In geology, the bedrock surface represents a regional unconformity whose surface expression varies from outcropping, to being buried by a variable thickness of geologically younger sediments that can range from a few centimetres up to several kilometres in depth. Owing to the often-significant differences in physical characteristics between bedrock and overlying unlithified deposits, DTB represents a key consideration of many geological, environmental, and geotechnical applications that are impacted by variations in near-surface sediment and rock properties. For example, thick and unconsolidated sediments host components of water-bearing, permeable strata that represent sources of potable groundwater, making information on DTB an important requirement for hydrogeological assessments at a regional or global scale [1]. Mapping spatial variations in DTB is valuable for the identification of sand and gravel deposits with aggregate potential, or for determining areas of thin sediment cover that represent suitable targets for mineral deposit exploration and development [2]. Regions with deeply buried bedrock can be subject to increased risks from geohazards particularly due to slope failures occurring in geotechnically soft/weak sediments, or from amplified ground shaking in earthquake-prone regions, making DTB a major factor within geotechnical and seismic hazard assessments [3]. Finally, information on DTB is a prerequisite to describe landscape-scale variations in numerous other surface properties such as run-off and sub-surface flow [4, 5], soil organic carbon stocks [6], and erosion/sedimentation [7].

The most common method for mapping spatial variations in DTB involves using geological picks from boreholes and spatial interpolation techniques to create an elevation surface of the bedrock. The total thickness of sediment is calculated by subtracting the interpolated bedrock surface from the ground elevation [8–11]. However, in areas without agricultural, urban, or industrial development, borehole data needed to support traditional spatial interpolations may be scarce. This can be especially challenging in regions with complex and/or rugged bedrock topography because interpolations based on sparse point data tend to produce overly smooth predictions that underestimate the bedrock elevation in areas with rapid terrain changes, leading to significant errors when calculating DTB [12, 13]. An alternative approach is to interpolate DTB directly based on the assumption that the bedrock surface closely follows the land surface [13–15]. However, this assumption does not hold true in intermontane valleys [16] and can result in unrealistic predictions in lowland areas [13]. Geophysical surveys using techniques such as high-resolution airborne resistivity have also provided detailed, continuous information about bedrock depth and near-surface stratigraphy [17–19]. However, these methods can be expensive to use over large areas and still require adequate borehole data for calibration.

Recently, information from digital elevation models (DEMs) and other remote sensing datasets has been used with statistical and machine learning models to understand spatial variations in the thickness and composition of surficial materials based on terrain and environmental characteristics. Studies using single-layer neural networks and ensemble tree methods have been used to predict DTB at regional [16, 20–22] and global scales [23]. However, most of these studies have achieved only moderate predictive accuracy (around 0.4–0.7 r^2^) or have been applied in regions with relatively shallow DTB, where the correlation to land surface topography is expected to be high. A potential limiting factor on predictive performance is that terrain and environmental variables may not be sufficient to explain spatial variations in DTB, and the concept of spatial autocorrelation, which is essential to many spatial interpolation methods, has not been incorporated into the machine learning process. Recent developments in machine learning approaches for spatial interpolation include using various spatial feature engineering approaches to potentially allow algorithms to develop rules that consider spatial location [24–27]. Because the prediction of deeply buried geological surfaces is likely to depend on the spatial location of the training points in addition to landscape relationships, incorporating geographic features into the modeling process may improve the predictive power of machine learning DTB models.

**Fig 1:**
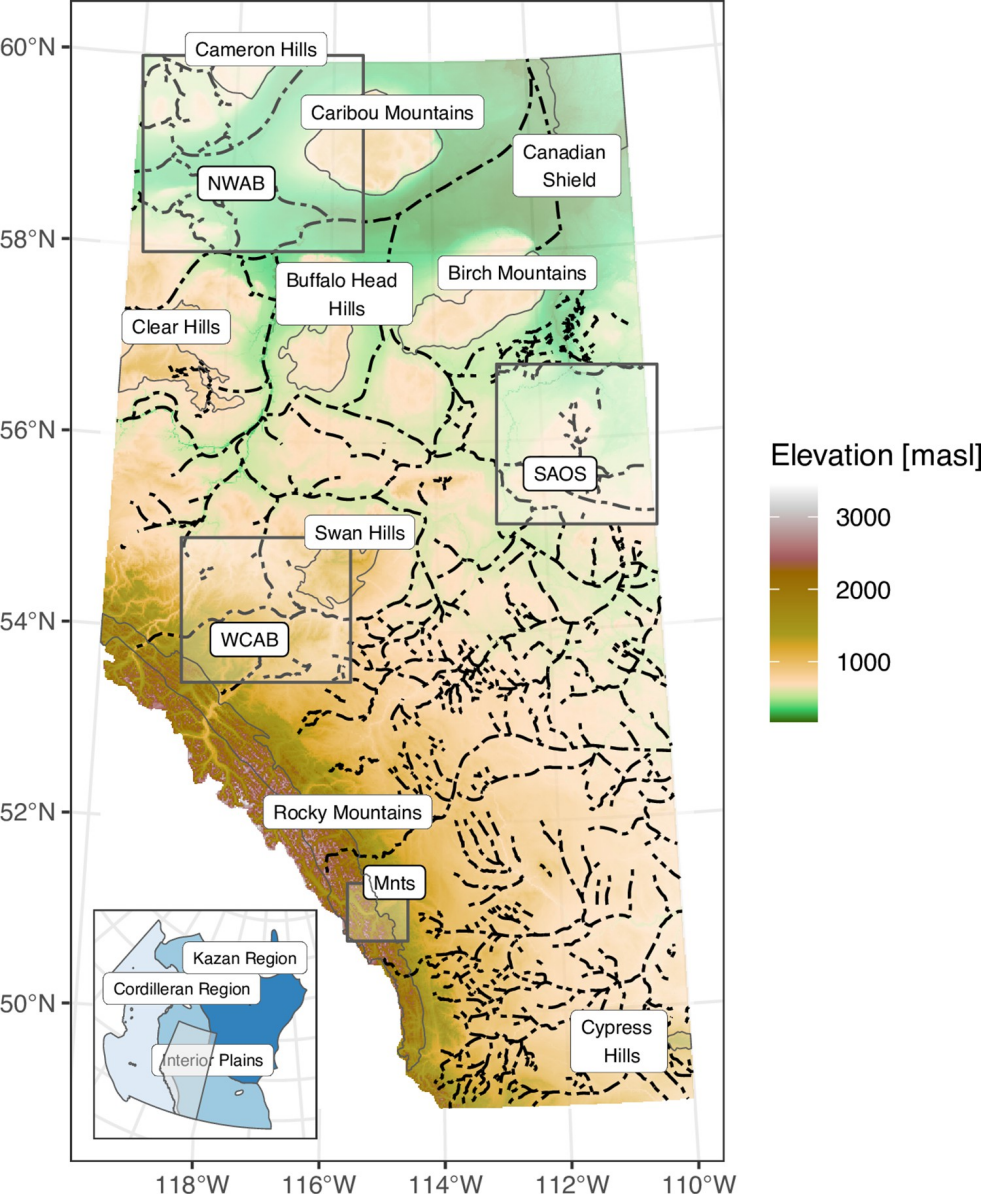
Topography of Alberta. Derived from the openly available ALOS DEM [28], displayed in metres above sea level (masl). Rectangular bounding boxes show the locations of the sub-regions that are used in the modelling experiments. Dashed lines show the thalwegs of major buried bedrock palaeochannels. The inset map shows the three physiographic regions of Canada that Alberta occupies [29].

In this study, we explore the use of machine learning approaches for mapping DTB in the Province of Alberta, Canada (Fig 1) using a large dataset of subsurface data and terrain predictors. We first developed a method to supplement existing bedrock topography picks using a natural language model to increase the spatial coverage of the training data. We then used several machine learning algorithms and different feature engineering strategies to model these training data, allowing the models to incorporate information about spatial autocorrelation and spatial structure of the training data. Finally, we evaluated the performance of these models in terms of their predictive accuracy and the geological plausibility of the predicted maps and derived bedrock topography surfaces.

## Regional setting

Alberta occupies ~662,000 km^2^ within the southern Interior Plains of Western Canada, with small portions occurring within the Canadian Shield and Western Cordillera (Fig 1). Alberta is host to diverse physiography that descends in elevation and relief from the Rocky Mountains and Foothills (3600–1000 m above sea level, masl) northeastward into the Eastern/Western Alberta plains (400–1500 masl) and Northern Alberta lowlands and plains (160–500 masl). Isolated uplands occur across Alberta and rise from 300–500 masl at their base to summits that range from 750–1400 masl. These include the Cypress Hills and Swan Hills in the southern and central regions of the province; the Clear Hills, Buffalo Head Hills, and the Cameron Hills in the northwest; the Caribou Mountains in the north; and the Birch Mountains in the northeast.

The thickness of sediments above bedrock varies substantially across Alberta, ranging from zero thickness (bedrock outcrops) to 400 m in depth within the eastern and northern parts of the province where a series of palaeochannel systems are filled with stratified and non-stratified sediments. Areas of thin sediment cover generally occur in the west, particularly in the Rocky Mountains and Foothills regions, although thin sediments also occur in two low-relief corridors through southern Alberta where Quaternary ice streams removed most pre-existing sediment.

## Materials and methods

### Data and preprocessing steps

#### Borehole and outcrop data

**Fig 2:**
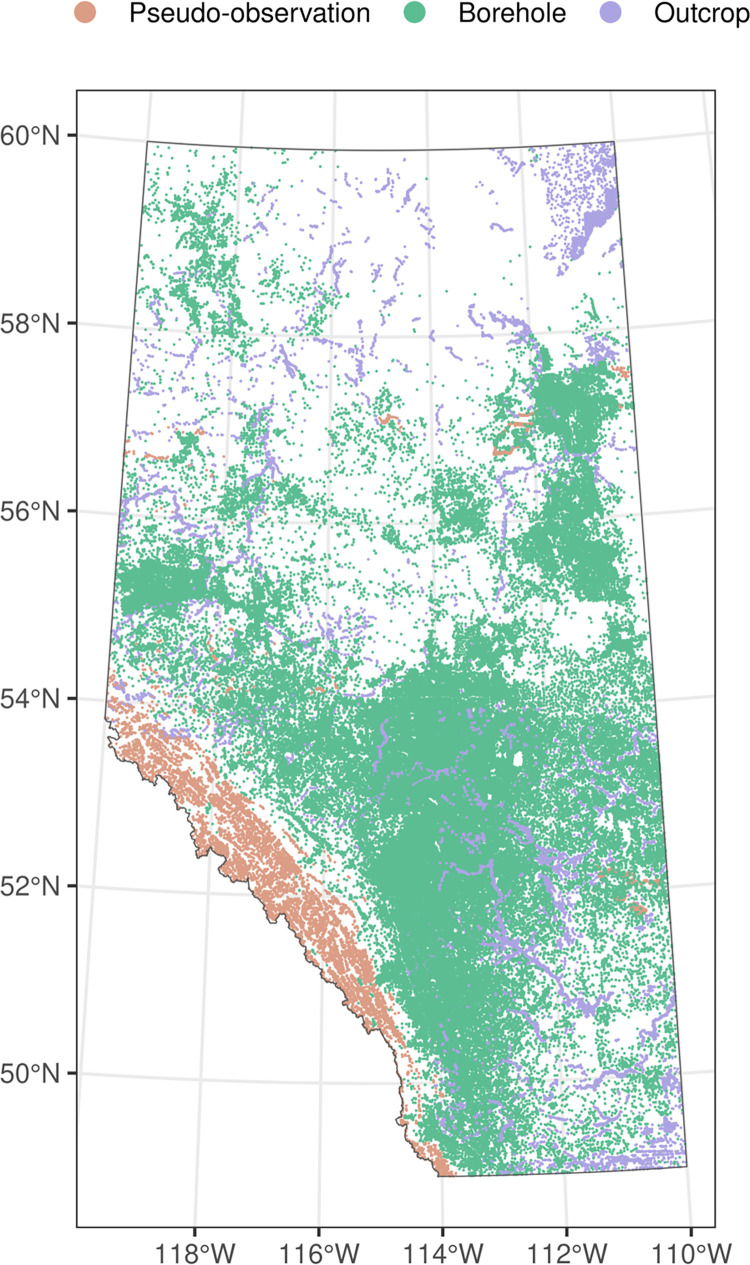
Distribution of the training data. Data locations are categorized by data source (borehole, outcrop, pseudo-observation).

The data used for mapping DTB was compiled from three sources (Fig 2) including:

1. Bedrock top picks made from geological boreholes (n = 153,443) with 74% being derived from water wells, 23% from oil and gas wells, and the remaining being derived from other sources such as geotechnical investigations, and mineral exploration boreholes. Further, another 162,220 previously uninterpreted water wells were used in the analysis.
2. Bedrock outcrop locations (n = 17,234) consisting of field observations, outcrops digitized from previously published geological maps and reports, and remotely interpreted observations using high-resolution satellite imagery.
3. Pseudo-observations (n = 7,911) of DTB derived from digitized contour maps of bedrock surface elevation but where the original data sources for the contour interpretations have not been explicitly captured. These included a small number of synthetically generated observations on steep-mountainous slopes (n = 990, DTB = 0) and points (n = 200) generated from the elevation of other subsurface units that are known to represent the uppermost bedrock unit.

#### Terrain and remote sensing datasets

**Fig 3:**
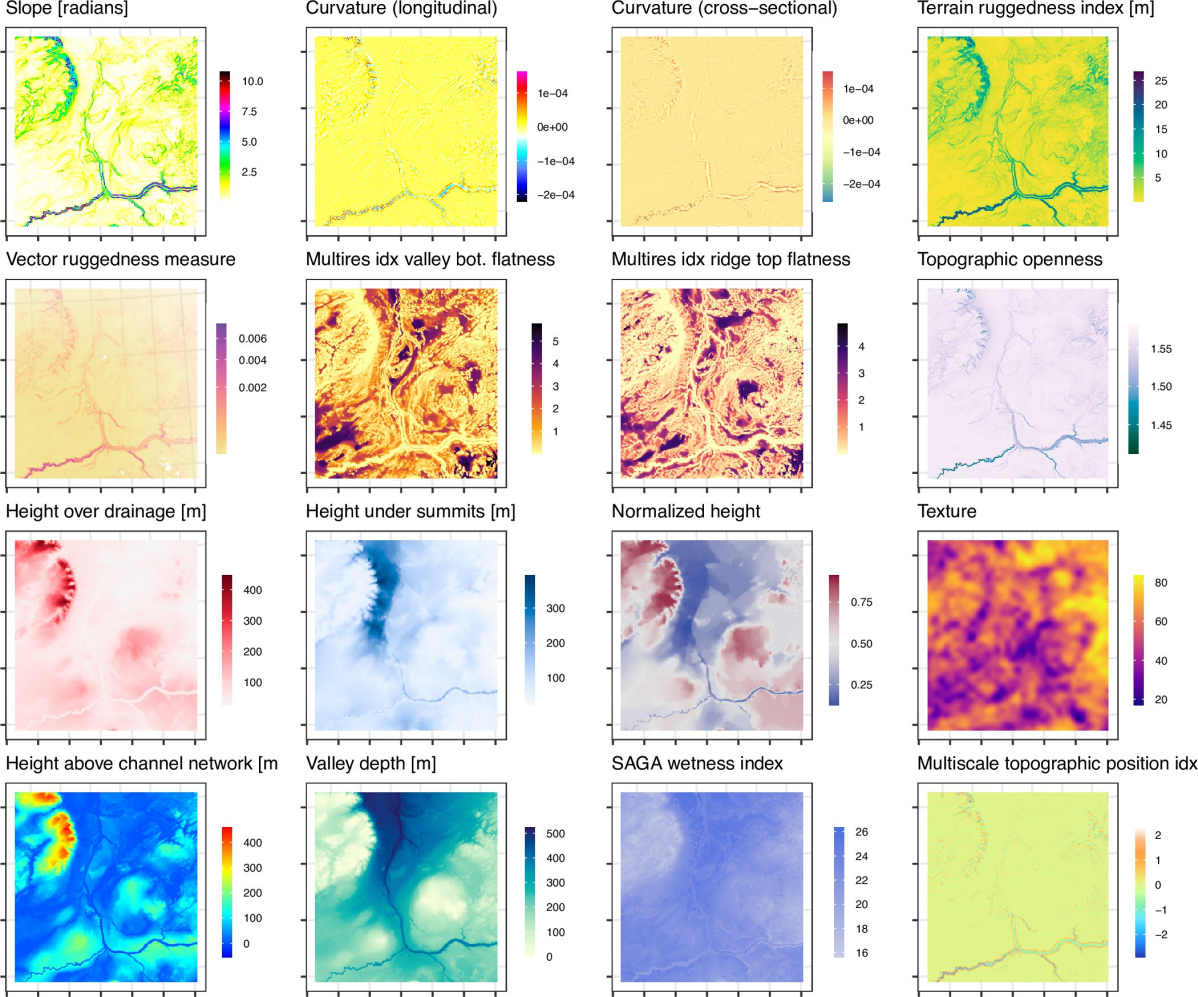
Examples of the topographic features for a region of NE Alberta. The displayed terrain ruggedness index and vector ruggedness measure were calculated using a 1-cell radius window.

A resampled version of the openly available ALOS DEM [28] at 500 m resolution was used as the primary source of topographic information for the study. This DEM was used to generate a suite of morphometric derivatives using the ‘Rsagacmd’ package [30] in the R statistical programming language in conjunction with the open-source SAGA-GIS software [31]. The morphometric parameters (Fig 3) broadly describe three types of relief, comprising:

1. Local morphology, based on standard terrain measures such as slope angle, curvature, and topographic roughness, which are calculated using relationships between pixels in locally moving windows across the DEM.
2. Regional morphology, based on calculations that model relationships between physiographic elements of the landscape using the DEM in its entirety, such as relative elevation differences to valleys and ridges, topographic openness, or the flatness of valley bottom areas.
3. Topographic wetness, based on relationships between slope angle and flow accumulation.

The derived terrain features were augmented with spectral data from a MODIS9A best pixel mosaic, created from early to late summer scenes during 2020. A summary of the features is provided in (Table 1).

**Table 1:** Terrain, geographic and spatial lag features used in the modelling process. The abbreviated feature names refer to the labels used in the figure. Some features occur in conjunction with integer suffixes such as ‘vdchn’ and ‘strahler’ which refer to the stream order used to calculate vertical or horizontal distances respectively.

Feature	Description
Channel base elevation (baselevel)	Topographic surface interpolated from river channel height
Digital Elevation Model (dem)	dem
Slope	Slope angle (radians)
Curvature (longc, crosc)	Horizontal/vertical components of slope curvature
Terrain ruggedness index (tri)	Absolute difference between pixel centre and neighbours
Vector ruggedness measure (vrm)	Variation in slope and aspect
Texture (texture)	Spatial density of pits and peaks in the DEM
Height under/over drainage/summits (ho, hu)	Height over drainage / under summits
Normalized height (nh)	Relative position between drainage and summits
Standardized height (sh)	Normalized Height multiplied by the DEM
Topographic openness (pos, neg)	Proportion of visible sky/ground
Multiscale topographic position index (mtpi)	Standardized difference between DEM cell and mean of neighbours
Multiresolution index of valley bottom flatness (mrvbf)	Relative flatness and lowness of valley floor areas
Vertical distance to channels (vdchn)	Elevation above channel base
Horizontal distance to channels (strahler)	Horizontal (planimetric) distance to river channels generated with the specified Strahlher order
Valley depth (vdepth)	Elevation below a surface interpolated from ridge lines
Relative slope position (rsp)	Relative height between channel and ridge elevation
SAGA Wetness Index (twi)	Modified topographic wetness index
X, Y coordinates	Grid coordinates

### Machine learning methods

Machine learning and data analysis were performed in the R statistical programming environment [32] using the ‘tidymodels’ suite of packages [33].

#### Natural language model of borehole lithologs

Despite the large data compilation efforts, some areas of the study region—specifically southeast Alberta and the Rocky Mountains and Foothills had limited data control (mostly shallow observations from outcrops, Fig 2). To avoid extrapolating the spatial predictions across these areas, a statistical natural language processing (NLP) model was used to automatically label previously uninterpreted water well lithologs that were abundant in these regions. NLP techniques are increasingly being used to extract information from geological text-based datasets, the results of which can then be used for geological modelling [34, 35].

The NLP model was trained upon the text descriptions of lithologs from compiled borehole dataset using a binary ‘bedrock’ or ‘surficial’ classification. Text descriptions (material type, description, and colour) for each logging interval were standardized by removing stop words and were tokenized using a bag-of-words approach. For lithological descriptions, only unigrams were retained because the ordering of the terms in the relatively simple water well descriptions did not convey much information (e.g., sand and silt, or silt and sand might be used interchangeably). For colour descriptions, bigram word sequences were used to retain terms such as ‘dark gray’ that are commonly used to describe units such as bedrock shale. Additional features were also added to the model based on simple text-matching patterns, as well as features that describe the number of intervals in the log, the maximum depth of the borehole, and the relative depth and relative thickness of the intervals.

For the NLP modelling, the XGBoost classifier was used [36], which is an ensemble decision tree-based algorithm that uses an additive, forward stepwise process where additional decision trees are fitted to correct the predictions from previous trees. XGBoost does this by using gradient descent to minimize the loss when adding new trees.

The predicted litholog intervals were then used to define the bedrock top based on the uppermost predicted bedrock interval that occurs beneath all surficial intervals. This approach, which ignores isolated intervals that are predicted as bedrock but overlie deeper surficial units, mitigates the problem that many Quaternary successions contain glaciotectonically displaced intervals of bedrock. The final predicted borehole DTB locations were used to augment the compiled borehole/outcrop dataset in regions where existing point-based estimates were sparse, only where no other observations were available within a 3 km radius.

#### Depth to bedrock spatial prediction

Three machine learning regression algorithms were evaluated for the DTB spatial predction: Random forests, XGBoost, and Cubist regression trees. The emphasis on using tree-based algorithms is based on their efficiency at scaling to large datasets and their popularity in previous spatial predictions, which reflects the theoretical capability of tree-based models to model spatial variations via recursive partitioning of the feature and geographic space [25].

Random forests (RF) [37] construct a ‘forest’ of uncorrelated decision trees using random subsets of the training data. This approach aims to reduce the overall variance of the predictions and mitigates over fitting by averaging predictions from many individual trees. In this study, we test both the original random forest implementation where the node splits in each tree utilize the most discriminative threshold, as well as the ‘extra trees’ configuration [38], which uses the best threshold from several randomly generated splits. The latter is computationally more efficient, produces a smoother regression model, and in some cases can lead to slightly improved predictive performances based on the increased diversity of the forest.

For the boosted tree algorithms, the previously described XGBoost model and a second boosted tree algorithm, Cubist [39, 40] were used in the spatial predictions. Cubist uses a different boosting approach where each tree corrects the predictions from the previous tree by subtracting the residual from the previous tree from the current tree’s prediction, summarized by:

$$y_{\left(m\right)}^{*}=y-(y_{\left(m-1\right)}-y)$$

where y^*^_(m)_ is the modified prediction for the current tree, and where (*y*_(m*-*1)_—*y*) is the residual from the previous tree. Cubist also differs in that it uses linear regression models at the terminal nodes of the tree, which allow the trees to extrapolate and can prevent trees from under-predicting the high and low tails of the data. An additional feature of Cubist is that the predictions can also be adjusted based on the values of neighbouring points in the feature space, with the weighting of neighbouring points using inverse distance weights.

For each model, the most pertinent hyperparameters that typically influence the fitting behaviour were optimized across a range of values using a grid search (for Random forests and Cubist) or randomized search (for XGBoost) approach (Table 2). For Random forests, these including the minimum number of observations to occur in a leaf-node (*min_n*), the number of predictors that are randomly available at each node split (*mtry*), and the criteria used in the decision tree splits (*splitrule*). For XGBoost, in addition to *mtry*, the maximum allowable depth of each tree (*tree_depth*) was tuned, along with the learning rate and the proportion of observations that are randomly sampled for each tree (*subsample*). Finally for Cubist, the number of boosting iterations (*committees*) and number of neighbours used in the accuracy adjustment (*neighbours*) were tuned.

**Table 2:** Hyperparameter tuning ranges for the machine learning models. The RF and Cubist models were tuned using a grid search with three levels, and the XGBoost model was tuned between the parameter range using a randomized search with 25 iterations.

Model	Type	Hyperparameters	Parameter Ranges
Random forests (RF)	Ensemble decision tree	min_n, mtry, splitrule	[1, 25], [5, 38], [variance, extratrees]
XGBoost	Gradient-boosted decision tree	max_depth, subsample, learn_rate, mtry	[5, 21], [0.6, 1.0], [0.05, 0.2], [5, 38]
Cubist	Boosted rule-based trees with linear regression terminal nodes	neighbors, committees	[0, 9], [10, 50]

#### Spatial feature engineering

Recently, there has been increasing interest in applying machine learning methods to spatial interpolation and a series of methods have been proposed based on introducing spatial features into the modelling process to incorporate information from the spatial structure of the training data. These spatial features include using: (a) x/y coordinates as features; (b) proximity measures to reference locations in the spatial domain, and (c) features derived from surrounding spatially proximal observations, either as spatial lag variables [26, 41–43] or using the values of neighbouring points directly as in the ‘RFSI’ approach [27]. For example, in the ‘RFsp’ method, Random Forest models were augmented by proximity measures using Euclidean distances to each sample point in the training data [25]. However, this is only computationally practical for small datasets [25], otherwise the number of generated features becomes extremely large (for our study, would involve adding ~ 200,000 predictors to the model, one distance measure per point, and would require > 450 GB RAM for the training data alone). In contrast, [24] demonstrated comparable results to regression kriging using only five Euclidean distance measures or ‘fields’ (EDFs) using arbitrary reference locations. Other studies have used proximities to geologically based reference locations, such as the edges of valley walls [16] or bedrock outcrops [44]. In contrast, using the values of neighbouring spatial point locations such as in the ‘RFSI’ approach [27] is conceptually more similar to traditional spatial interpolation techniques and was shown to outperform the RFSp approach in some datasets.

To find the best spatial feature engineering approach for the DTB spatial prediction, each of the tested machine learning algorithms was trained using five different sets of features, consisting of:

1. **feature set T**, containing terrain and MODIS data only without any additional features being introduced to specifically account for spatial dependence.
2. **feature set T+CRDS**, where x/y coordinates are added to the first feature set.
3. **feature set T+EDFs**, containing the terrain and MODIS data, augmented by the EDFs approach using distances to five reference locations (corners and centre of model domain) [24].
4. **feature set T+NN**, where the target values and distances to neighbouring point locations are used directly as additional features.
5. **feature set T+SL**, where spatial lag variables consisting of the distance weighted mean of surrounding data points are used as additional features.

For the spatial lag variables, the k-nearest neighbouring points to each training and/or prediction point were queried using the fast kd-tree search method. Although many new features and summary statistics could potentially be extracted from both the response and predictor variables, for parsimony we focused on three features using 5, 10, and 15 neighbouring points. The weighted mean of the response variable y of these points was then calculated by:

$$y_{i}^{*}=\frac{\sum_{j=1}^{k}W_{j}y_{j}}{\sum_{j=1}^{k}W_{j}}$$

where y*_i_ is the prediction point and *j* = 1,2,3 … *k* is each neighbour. The weights of the neighbours were assigned based the probability density of a Gaussian kernel to provide smooth aggregations of the surrounding points:

$$W(x_{i},x_{j})=\frac{1}{(\sigma \sqrt{2\pi )}}exp\left(-\frac{d(x_{i},x_{j})P{\left(1/2k\right)}^{2}}{2\sigma^{2}}\right)$$

where Gaussian probability density function has a standard deviation σ = 1 and a mean of zero, and where *d*(*x*_*i*_−*x*_*j*_) are the row-normalized Euclidean distances between points *i* and *j*, which are weighted by the quantile of the Gaussian distribution that is equal to the 1/2*k* [45]. This uses the concept that when *k* is large, the distances are scaled across a greater width of the Gaussian distribution, which will make the prediction more sensitive to neighbours near the prediction point, and when *k* is small, the distances are scaled across a narrower width of the distribution, amounting to a more equal weighting that will smooth and reduce the variance of the prediction. We note that a wide range of other weight functions could potentially be used or optimized as a hyperparameter.

Finally, to understand how these augmented spatial features were influencing the models, feature importance scores were calculated for each approach using a permutation method based on the ratio in r^2^ scores obtained from predictions using the original model, and predictions obtained after each variable is permuted. These scores are presented as relative scores based on dividing each score by the score of the most important feature for each feature set and model.

#### Model validation

10-fold cross-validation was used throughout the modelling process to provide unbiased estimates model performance. For the spatial prediction models, an inner (nested) 3-fold cross-validation was also used to optimize the model’s hyperparameters. Model performances were summarized and compared based on:

- The RMSE scores on the out-of-fold samples during cross-validation to provide a global estimate of model error.
- For the spatial prediction models, the local Moran’s I statistic as calculated on the residuals of the out-of-fold samples was used to quantify the degree of residual spatial autocorrelation in the predictions. Local Moran’s I [46] is a local indicator of spatial association (LISA) that describes the spatial pattern of a feature, in this case, the model’s residuals, based on the slope of a regression between a feature and its spatially lagged values. Rather than displaying the Moran’s I directly, point locations that have statistically significant I-values are classified according to the quadrants of the Moran’s scatterplot, describing whether each point shows positive or negative spatial autocorrelation. Positive spatial autocorrelation in the residuals occurs where a point has high residual and is surrounded by neighbouring points with high residuals (High-High), or conversely where a point has a low residual and is surrounded by neighbouring points with low residuals (Low-Low).

The evaluation of the spatial prediction approaches was performed in four sub-regions of the province (Fig 1), consisting of two lowland sub-regions with thick deposition sequences (NWAB, SAOS), an upland area having moderate variability in DTB (WCAB), and a fourth region with mountainous terrain (MNTS). The predicted maps from the different combinations of algorithms and spatial feature engineering techniques were also evaluated qualitatively in terms of the quality and geological plausibility of the spatial predictions.

Finally, after the optimal machine learning approach was selected, the model was retrained on the full dataset to spatially predict DTB across the province. To place this prediction in context, the results are compared with DTB maps obtained from the two most popular interpolation approaches used in DTB / bedrock topography modelling: inverse-distance weighted (IDW) and ordinary kriging (OK). For the IDW/OK interpolations, two strategies were used as benchmarks where: (a) DTB is interpolated directly using ordinary kriging (OK-DTB), and (b) bedrock elevation surface elevation is interpolated directly using IDW (IDW-BSE). IDW was used to interpolate bedrock elevation as the response variable because the data displayed strong spatial non-stationarity and could not be modelled with a single variogram across such diverse terrain (mountains, uplands, and plains). IDW interpolation used a weighted average from 16 neighbouring points with a distance-weighting power of 2. The OK interpolations used variogram models that were fitted using the R ‘gstat’ package [47] using an exponential model Kriging interpolation was performed in a local neighbourhood using the closest 16 points.

## Results

### Natural language model classification performance

**Fig 4:**
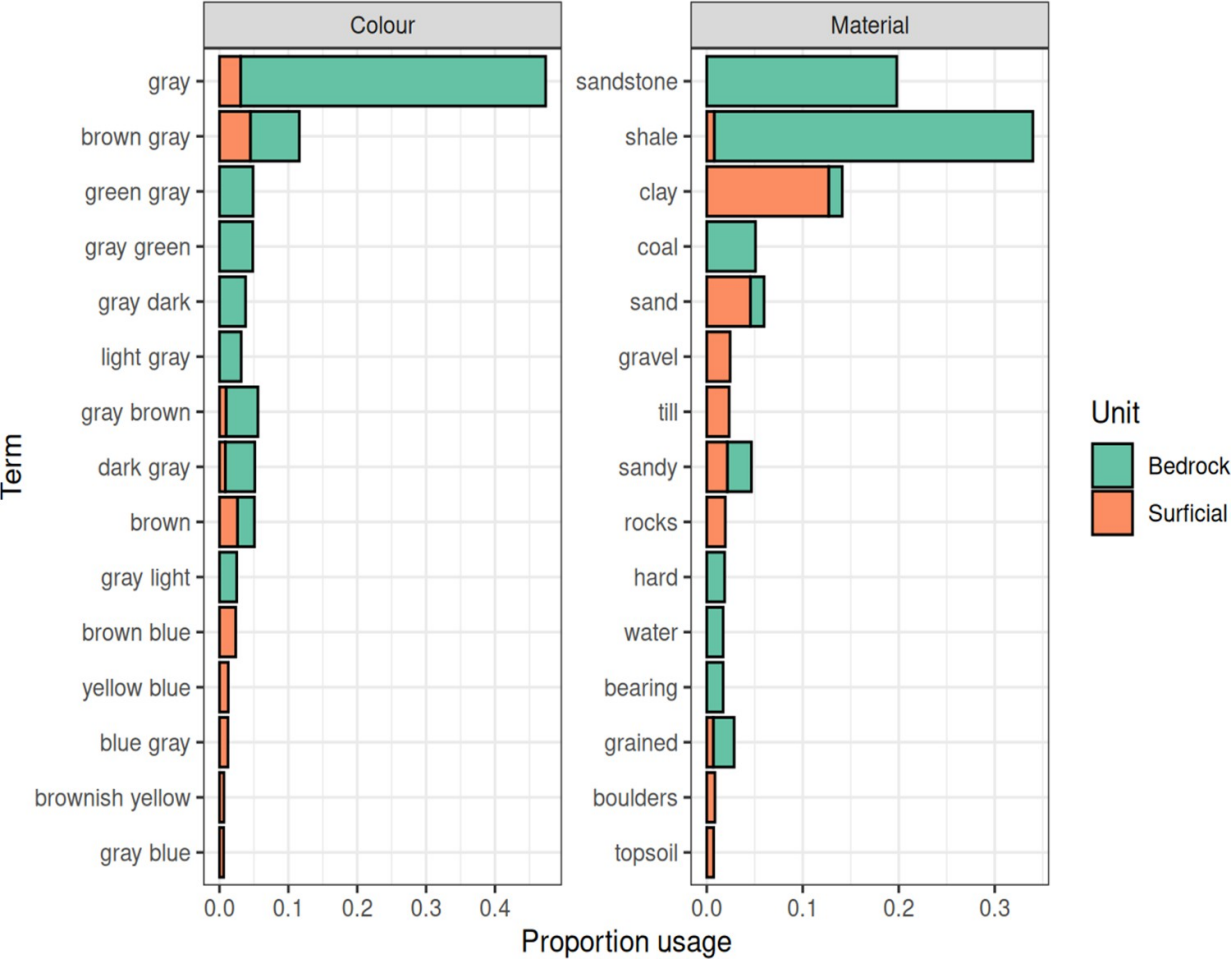
Word frequency plots. Common lithological and colour descriptions associated with bedrock and surficial litholog descriptions. The width of the bars displays the proportion of word usage associated with surficial and bedrock units. These terms are split into those that relate to lithology, and those that relate to colour.

Word frequency plots (Fig 4) show the relative abundances of geological terms in the cleaned/standardized litholog interval descriptions that are associated with bedrock and surficial units. Expected terms such as ‘shale’ and ‘sandstone’ characterize bedrock litholog descriptions, and terms such as ‘clay’, ‘till’, ‘gravel’ and ‘rocks’ are commonly used in surficial unit descriptions. Other terms such as ‘coal’ occur more commonly in bedrock intervals, but also in surficial intervals (coal is commonly assimilated in glacial deposits). Colours do not appear to provide such a strong separation between surficial and bedrock units, except that bedrock intervals are overall described as ‘grey’, while surficial intervals include a mixture of ‘brown’ and ‘grey’ terms.

**Fig 5:**
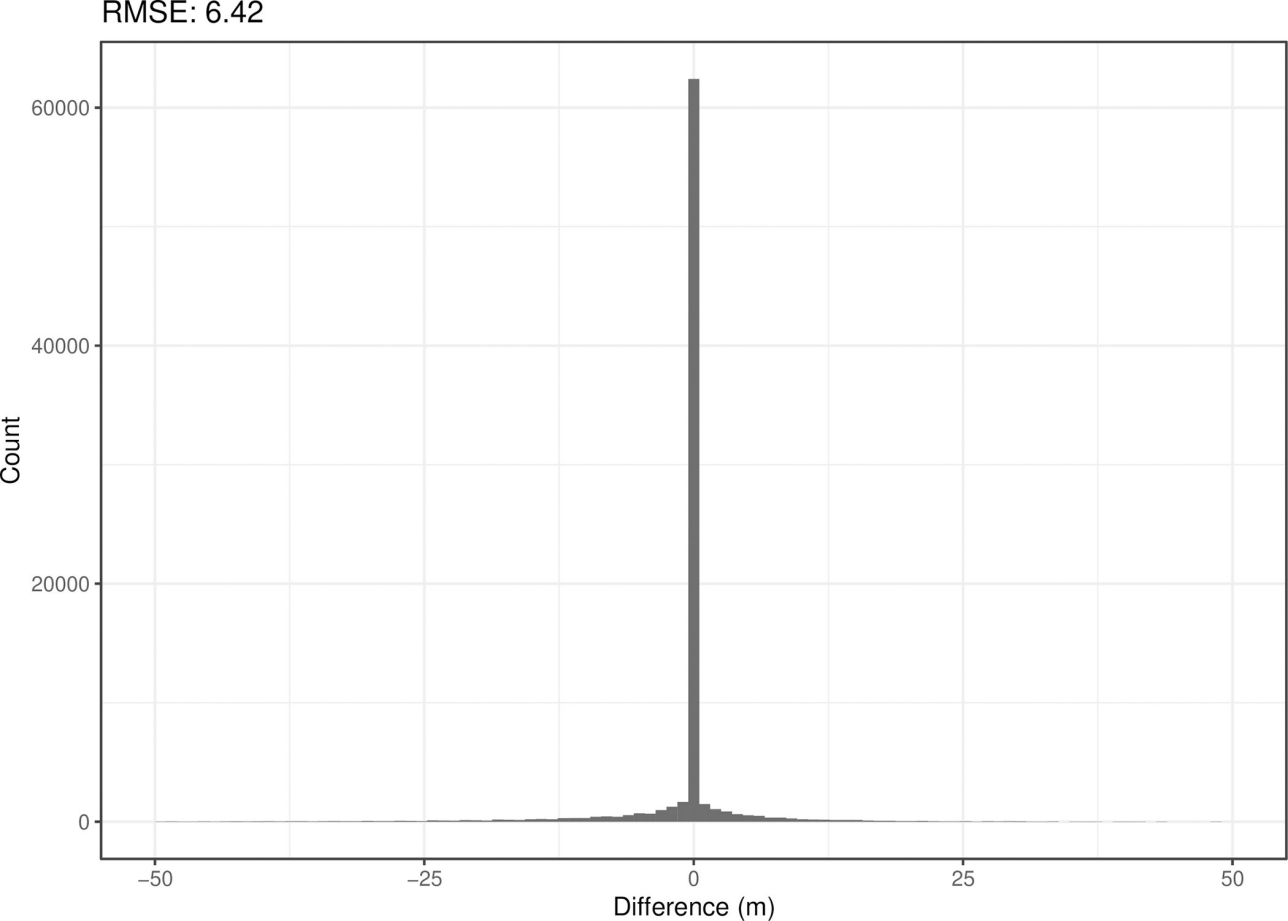
Histogram of the residuals of the NLP model. Shows the differences/residuals (true bedrock depth—predicted bedrock depth) obtained on the test set.

The predictive performance of the model as evaluated during cross-validation was high (RMSE = 6.42) (Fig 5). Bedrock top depths from the remaining water wells were predicted in the data poor regions of the study area using the trained NLP model and were appended to the compiled training dataset.

### Depth to bedrock spatial prediction

#### Predictive performances

**Fig 6:**
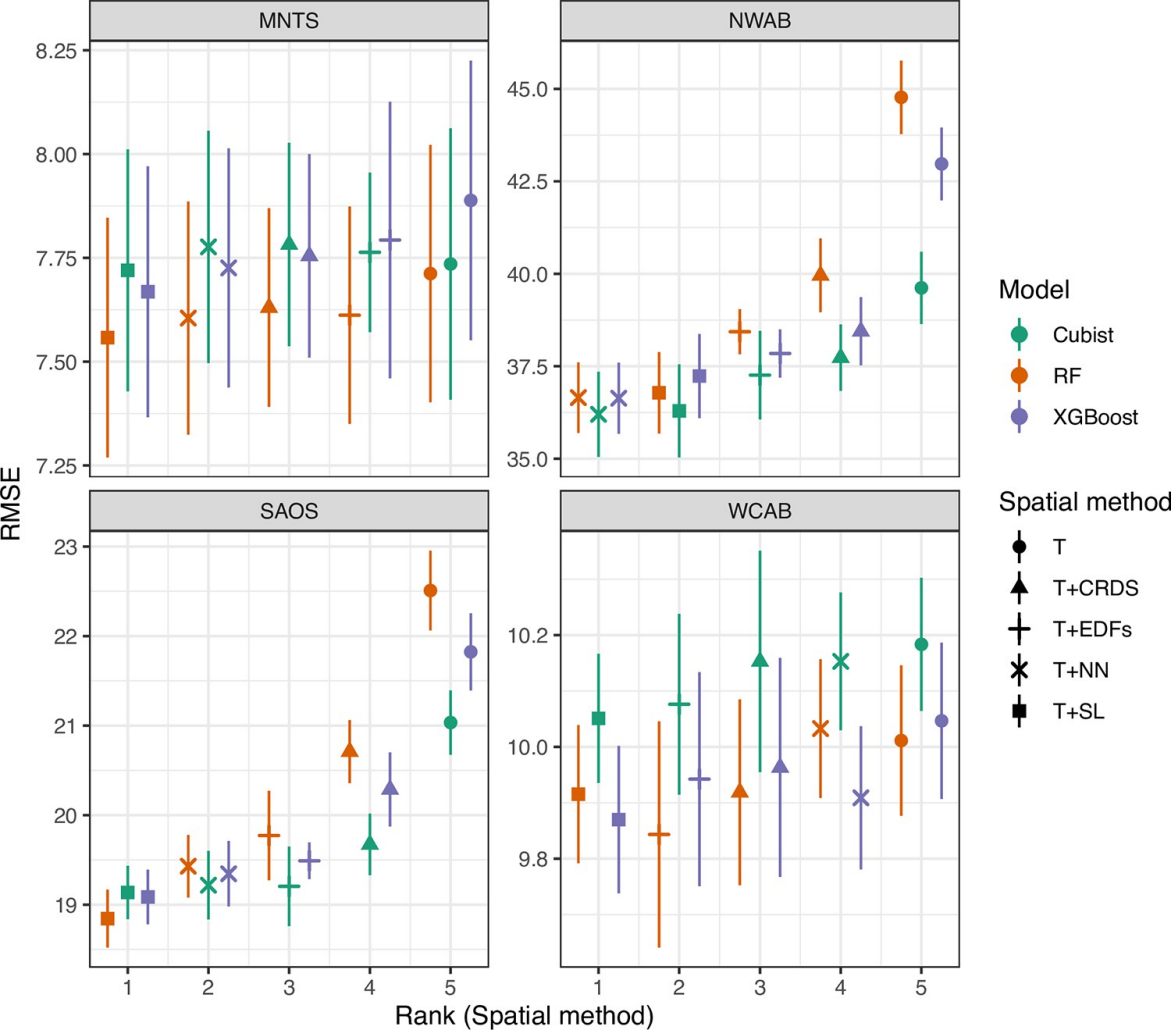
Point range comparisons of RMSE. Mean and standard errors for the tested models and feature sets for each tested sub-region. The points are ordered on the x-axis based on the rank of the overall scores for each spatial feature engineering method, per sub-dataset.

Fig 6 shows the RMSE scores resulting from the 10-fold nested cross-validation. The choice of modelling algorithm does not appear to significantly affect predictive performance in most of the sub-datasets. However, the choice of spatial feature engineering approach shows a more significant effect; models trained using only terrain and MODIS features (feature set T) are overall ranked last in all sub-datasets, albeit the differences are small in the two upland and/or mountainous regions. The inclusion of coordinate or Euclidean distance features into the models (T+CRDS, T+EDFs) supplies moderate performance benefits. The spatial neighbours and/or spatial lag methods (T+NN, T+SL) are ranked first, with the spatial lag approach (feature set T+SL) being ranked first in three of four sub-datasets (MNTS, SAOS, WCAB), and being equivalent to the top ranked T+NN method in the NWAB sub-dataset.

To further examine the size of the improvement in predictive power for the spatial feature engineering approaches, a post hoc Bayesian ANOVA model was used to estimate the posterior distributions for the performance metrics. The Bayesian ANOVA model considers that the relatively performance of each method tends to be similar across each cross-validation resample, i.e., there is a strong resample-to-resample covariance. This information is lost when comparing the models based on summarized means/standard errors alone, which under-powers the ability to detect differences between the methods. The Bayesian ANOVA approach uses a random effects linear model to account for the resample-to-resample effects, where a random intercept term is drawn (with 5000 repeats) from a t-distribution [48]. From these distributions, the probability that the overall top-performing spatial feature engineering method (T+SL) is equivalent to the other methods is assessed.

For brevity, we focus on the RF model because the choice of regression tree algorithm has negligible effect in these datasets. From the posterior distributions of the RMSE scores from the Bayesian linear model fits (Table 3), the best performing feature engineering method (T+SL) shows a high probability (> 0.9) of reducing the RMSE (compared to a zero reduction) in the NWAB and SAOS sub-datasets when compared to most of the other spatial feature engineering methods, apart from the T+NN approach which has similar performance. In contrast, in the two upland and mountainous sub-datasets, the T+SL method is equivalent to the other methods.

**Table 3:** Reductions in RMSE from the posterior estimates of the Bayesian ANOVA model for pairs of models. Negative values occur when the reference modelling method, Random forests with the T+SL feature set reduces the RMSE score, and positive values occur where the RF (T+SL) method increases the RMSE compared to the other model. The probability column describes the probability that the RMSE scores of the reference method are different compared to the other model. The bolded text shows the model pairs that have a high probability (> 0.9) of being different.

Reference Method		Estimates	
	Comparison Method	Difference (RMSE)	Probability
MNTS			
RF (T+SL)	RF (T+CRDS)	-0.06	0.54
RF (T+SL)	RF (T+EDFs)	-0.05	0.53
RF (T+SL)	RF (T+NN)	-0.04	0.52
RF (T+SL)	RF (T)	-0.13	0.57
NWAB			
RF (T+SL)	**RF (T+CRDS)**	**-3.18**	**1.00**
RF (T+SL)	**RF (T+EDFs)**	**-1.66**	**0.99**
RF (T+SL)	RF (T+NN)	0.12	0.44
RF (T+SL)	**RF (T)**	**-8.00**	**1.00**
SAOS			
RF (T+SL)	**RF (T+CRDS)**	**-1.86**	**0.99**
RF (T+SL)	**RF (T+EDFs)**	**-0.92**	**0.90**
RF (T+SL)	RF (T+NN)	-0.58	0.78
RF (T+SL)	**RF (T)**	**-3.67**	**1.00**
WCAB			
RF (T+SL)	RF (T+CRDS)	-0.00	0.50
RF (T+SL)	RF (T+EDFs)	0.07	0.46
RF (T+SL)	RF (T+NN)	-0.12	0.57
RF (T+SL)	RF (T)	-0.10	0.55

#### Feature importance scores

**Fig 7:**
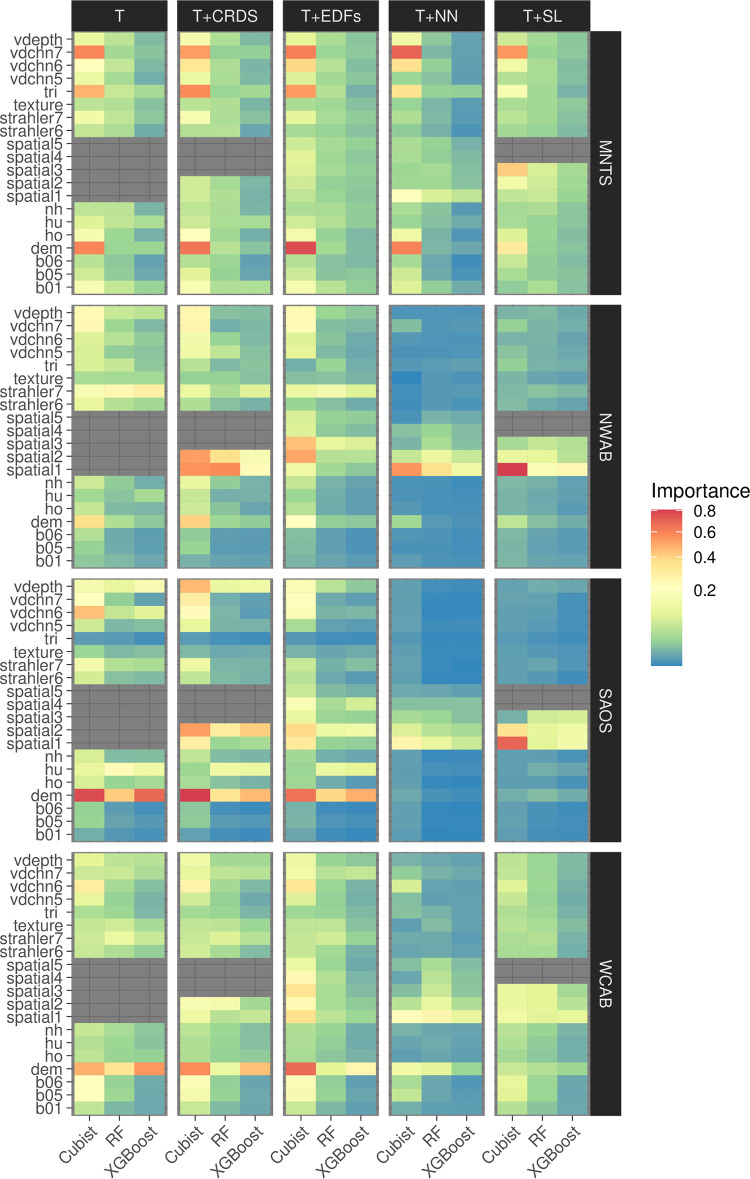
Relative feature importance scores. r-squared scores normalized relative to the top scoring feature. Features with low importance scores (< 1%) are omitted from the plot. The features spatial1-5 refer to the top spatial variables for each method, such as x, y coordinates, the five EDF variables, the closest five neighbouring points, or the spatial lag variables using 5, 10 and 15 neighbours.

Heatmaps of the feature importance scores for the overall top 20 most important features (Fig 7) show that the importances are region-dependent, but within each region, broad similarities in the rankings occur between the different modelling methods.

In general, features that describe regional variations in relief and physiographic setting, such as valley depth, elevation/baselevel and height relative to drainage and ridge positions, are highly influential in all the models and sub-regions. The importance of features such as valley depth and proximity to channels is consistent with the physiographic setting of Alberta, where many rivers incise into bedrock and the landscape is fundamentally controlled by fluvial erosion and deposition [49]. However, the mountainous sub-region differs in that spectral features and local relief (such as tri1) also have high importances, due to the substantial amounts of exposed rock surfaces and varied terrain.

When spatial features are included in the models, coordinate features are important in feature sets T+CRDS and T+EDFs. In contrast, when EDFs are used, only moderate importances are assigned to them by the algorithms. In feature sets T+NN and T+SL, spatial features that utilize information from neighbouring points are ranked as highly important, and this is also accompanied by a significant reduction in the relative importance of the terrain/spectral features in the two lowland sub-datasets (NWAB, SAOS), but less so in the two upland areas.

#### Residual spatial autocorrelation

**Fig 8:**
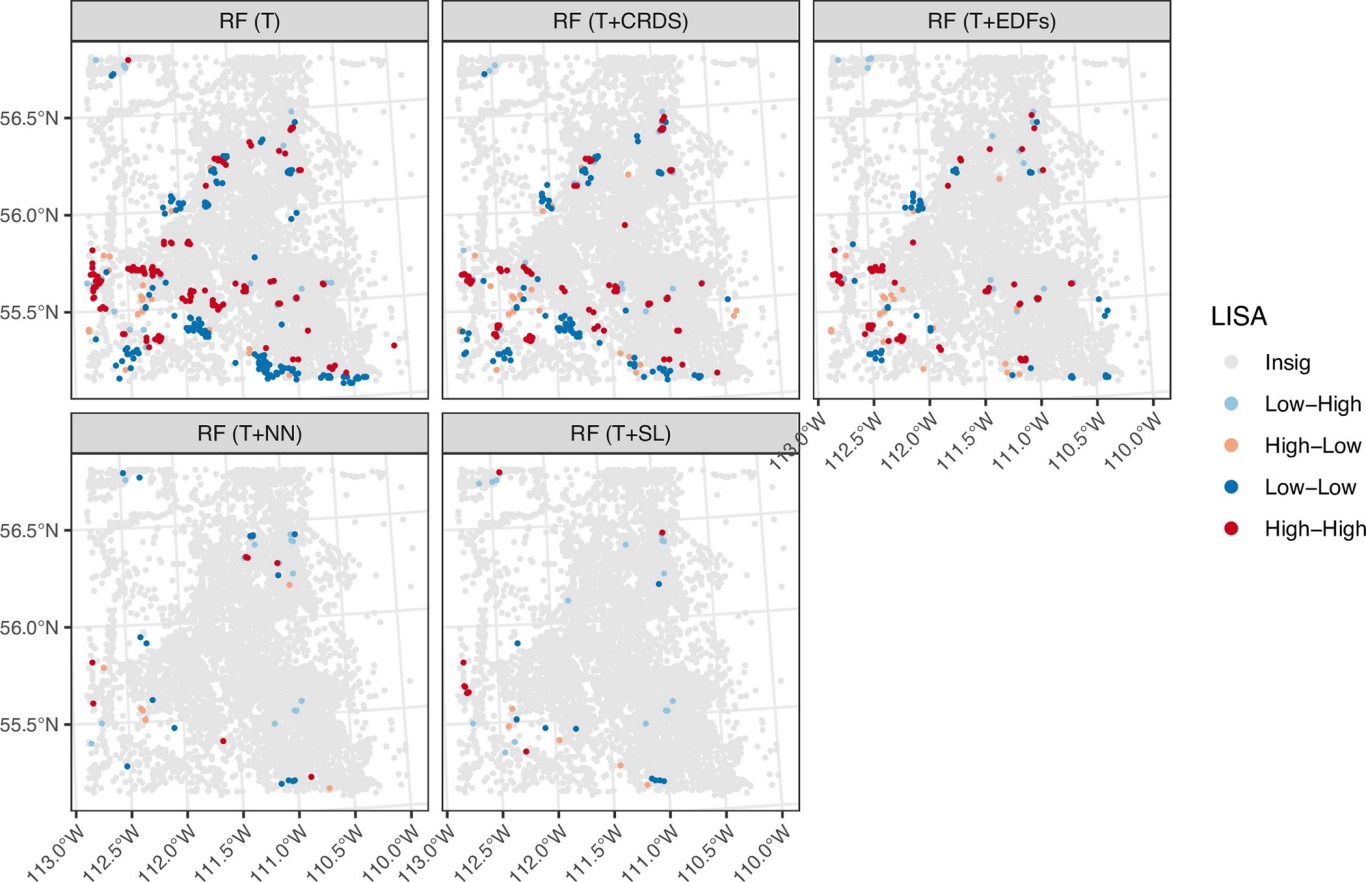
Local indications of spatial association (LISA). Examples of LISA statistics shown for three feature sets (T, T+EDFs, T+NN, T+SL) within the SAOS sub-dataset using the RF model. These feature sets show that the spatial lag feature engineering approach reduces the degree of spatial autocorrelation in the predictions compared to when only terrain/spectral features are used in the modelling.

Fig 8 shows the spatial distribution of local association of the model’s residuals, focusing on the RF model predictions in the SAOS sub-dataset as an example. The LISA maps show many clusters of positive spatial autocorrelation when only terrain/spectral features are used in the modelling (feature set T). The extent of positive spatial autocorrelation is reduced when the T+NN or T+SL feature engineering approaches are used but are only slightly reduced based on feature sets T+CRDS and T+EDFs.

#### Comparison of the predicted maps

**Fig 9:**
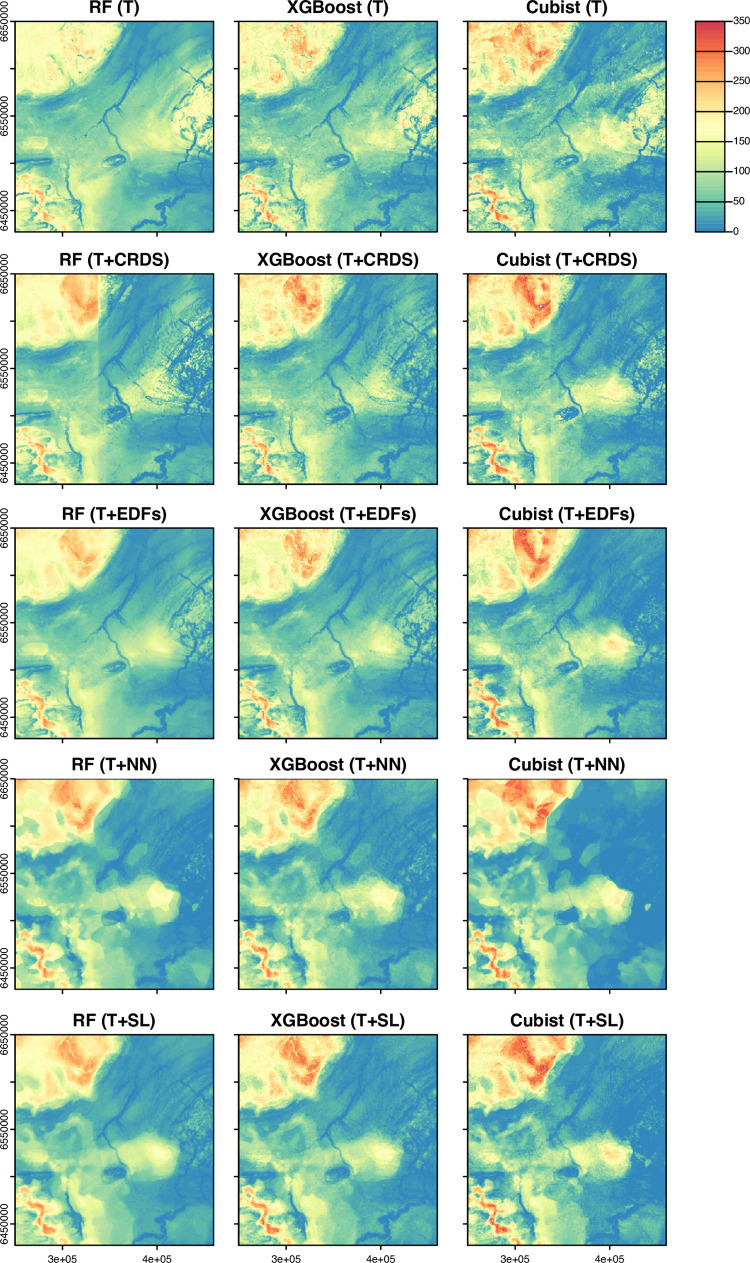
Predicted depth to bedrock (DTB) maps. Shown for the Random forests, XGBoost and Cubist machine learning models and feature sets.

**Fig 10:**
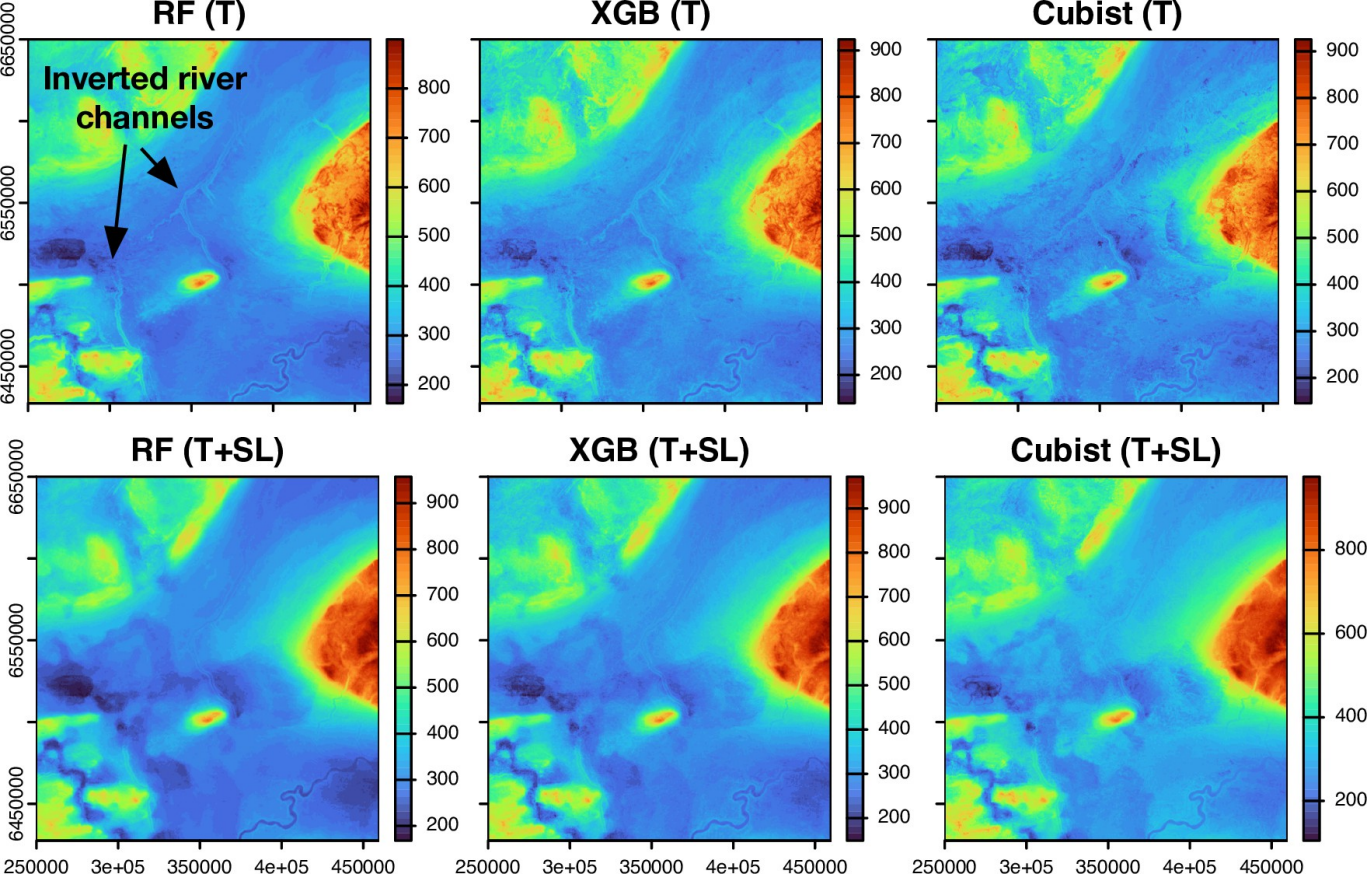
Derived bedrock topography maps. Examples of derived bedrock elevation surfaces are shown for the machine learning models, using the terrain-only (T) and spatial lags (T+SL) feature sets as examples.

Fig 9 provides a comparison of the predicted maps from the different feature engineering approaches and models using the NWAB sub-region as an example. Spatial variations in DTB in this area are driven by the occurrence of thick sediment infills associated with a buried palaeochannel network (Fig 1) and thin sediment occurring along the steeper flanks of the uplands and the margins of incised river channels. Fig 10 also provides examples of associated bedrock topography surfaces as derived by subtracted the DTB estimations from the DEM.

Although the predicted maps share broad similarities, maps from the boosted tree methods are characterized by a high degree of local variability, which gives the DTB and derived bedrock topography maps an overly rough/noisy appearance. Furthermore, none of the algorithms adequately predict the local variations in DTB that are associated with the buried palaeochannels or incised valleys when using either the terrain (T) and/or coordinates (T+CRDS) feature sets. The use of coordinate features alone also leads to a ‘box-like’ pattern to occur in some of the predictions caused by decision tree splits in the x, y coordinates, making this approach potentially unsuitable for spatially predicting DTB. These maps also display other spurious spatial artifacts that occurred around incised river valleys, which display an inverted appearance due to a halo of thicker sediment being predicted along the channel margins Fig 10.

The delineation of the channels is partially improved when using the T+EDFs feature set. However, some of these maps contain circular spatial artifacts caused by an abrupt change in the predictions at specific distances in the Euclidean distance features. The most detailed and realistic representation of the palaeochannels occurs in maps based on methods that use information from neighbouring point locations in the T+NN and T+SL approaches, and the unrealistic halos around incised channels are also largely removed. However, when using the neighbouring points directly as in the T+NN approach, a strong Thiessen polygon-like structure is present in the predictions. Based on the feature importance scores, this is likely caused by the dominance of the closest neighbouring point, which without distance weighting, causes the model to behave like a 1-nearest neighbour interpolation. Consequently, this spatial feature engineering approach is not considered appropriate for predicting DTB. In contrast, the maps predicted using the T+SL feature set consistently yield the most geologically plausible results because they are free of rapid, step-like changes and other spurious patterns, but capture the detail of complex geological features such as the narrow palaeochannels.

#### Application to provincial-scale modelling

**Fig 11:**
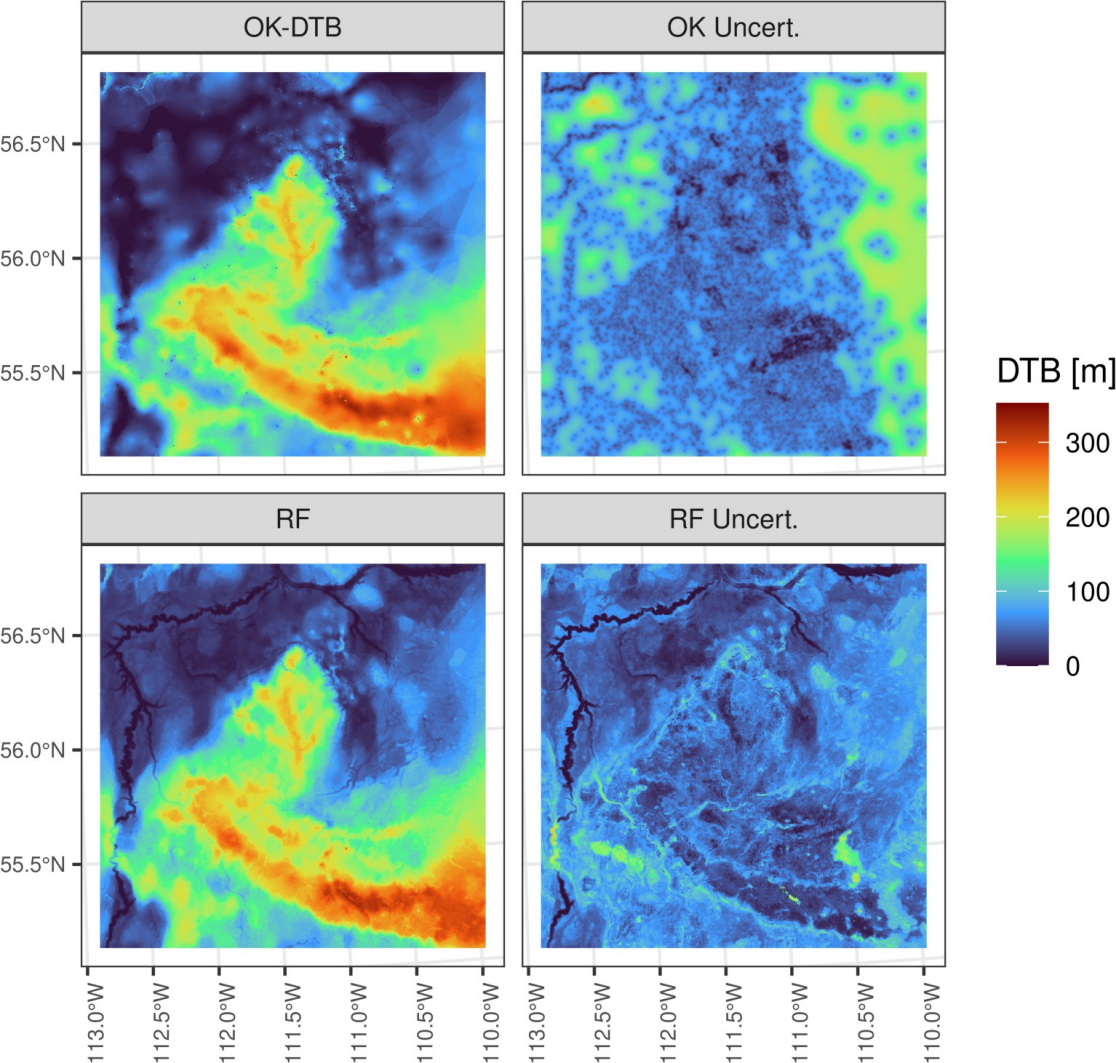
D bin plots of observed and predicted observations. Cross-validation results for the final model predictions for the region of Alberta using the selected RF model and T+SL feature set, compared to a 1:1 relationship (dotted line). Each rectangle represents a binning of the observed and predicted values, and the colour fill represents the number of observations in the bin. The solid line is from a linear model fitted to the observed vs. predicted values.

From the first assessment, the spatial lag feature engineering approach using the RF model was selected for the final DTB prediction at a provincial scale. The resulting RMSE scores show that the RF model has similar overall scores compared to ordinary kriging (Table 4). The intercepts of a linear regression fitted to the observed and predicted observations (Fig 11) are slightly higher than zero for both RF and OK-DTB, showing that both approaches have a slight tendency to underestimate DTB at large values and overestimate DTB at very shallow values. The difficulty of predicting the tails of a distribution is well known for RF and results from the random process in the decision trees causing a small portion of trees in the ensemble to always produce an erroneously low or high prediction.

**Table 4:** RMSE scores for the final spatially lagged RF approach as applied to the provincial dataset. For comparison, the RMSE scores obtained from ordinary kriging of depth to bedrock (OK-DTB) are also shown, along with the intercept and slope of linear models fitted to the truth vs. predicted cross-validation results.

Model	RMSE	Standard Error	Intercept	Slope
RF	13.8	0.09	3.26	0.90
OK-DTB	13.6	0.09	2.82	0.91

**Fig 12:**
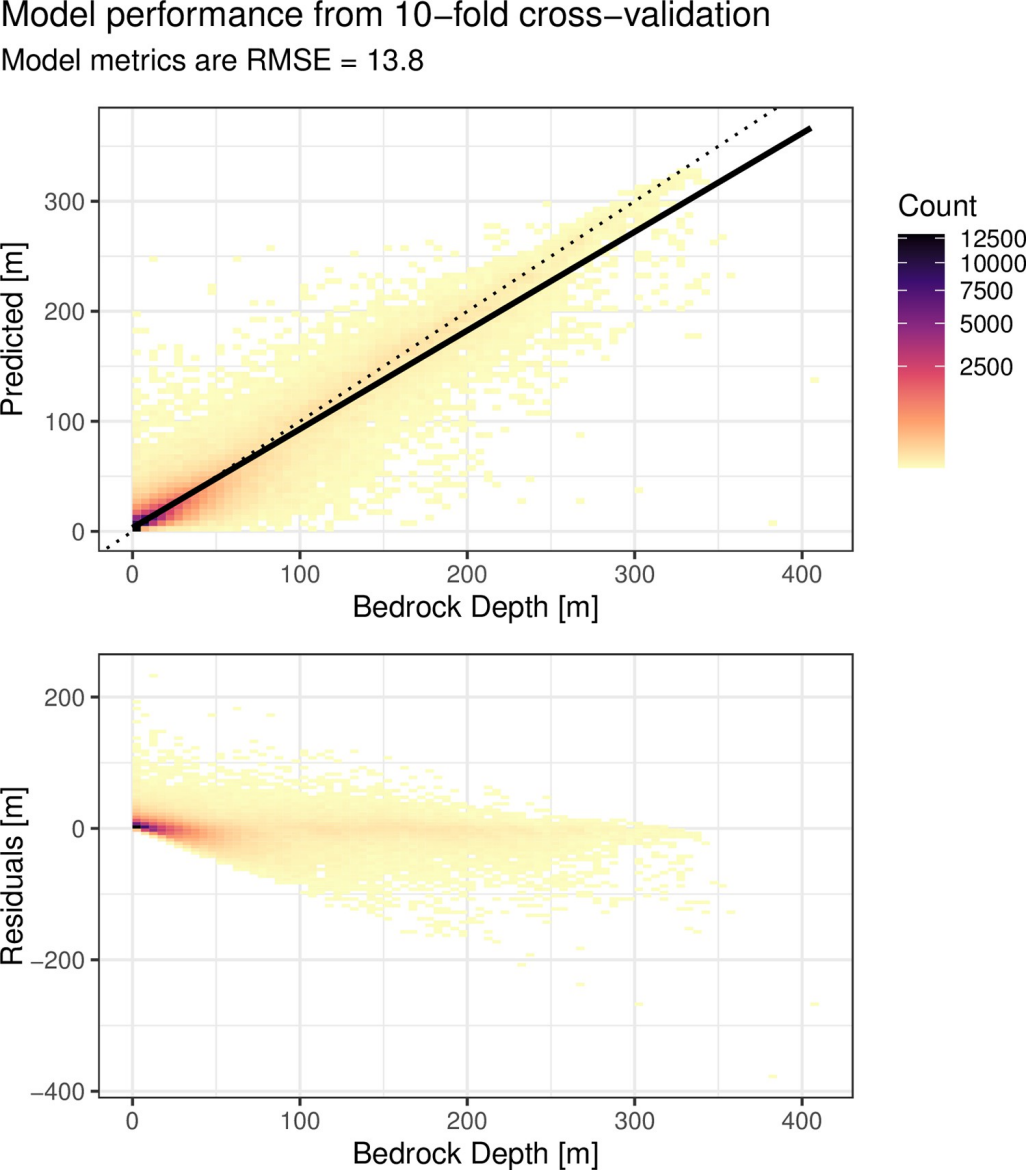
Comparison of the predicted DTB and uncertainties. The range of the p90—p10 prediction interval from the RF model with spatial interpolations of DTB using ordinary kriging (OK-DTB) in the SAOS region. The increased detail and more realistic prediction of DTB variations around major physiographic features such as river valleys and local uplands is evident in the RF results.

Despite similar performances, comparison of the RF predictions to that produced by the OK-DTB method shows that the machine learning approach captures distinct aspects of the data (Fig 12). In particular, the increase in detail in the machine learning predictions is evident in areas of incised terrain such as in major river valleys, where interpolating DTB using the ordinary kriging leads to near-constant DTB estimates within incised areas, and several major geomorphic elements of the landscape are completely absent in the predictions, making them geologically unrealistic. Similarly, comparison of the uncertainty estimate maps from the two methods also shows that the RF uncertainty map is more detailed and informative by highlighting several areas of terrain where there is a large uncertainty in DTB, for example around the flanks of a local upland.

**Fig 13:**
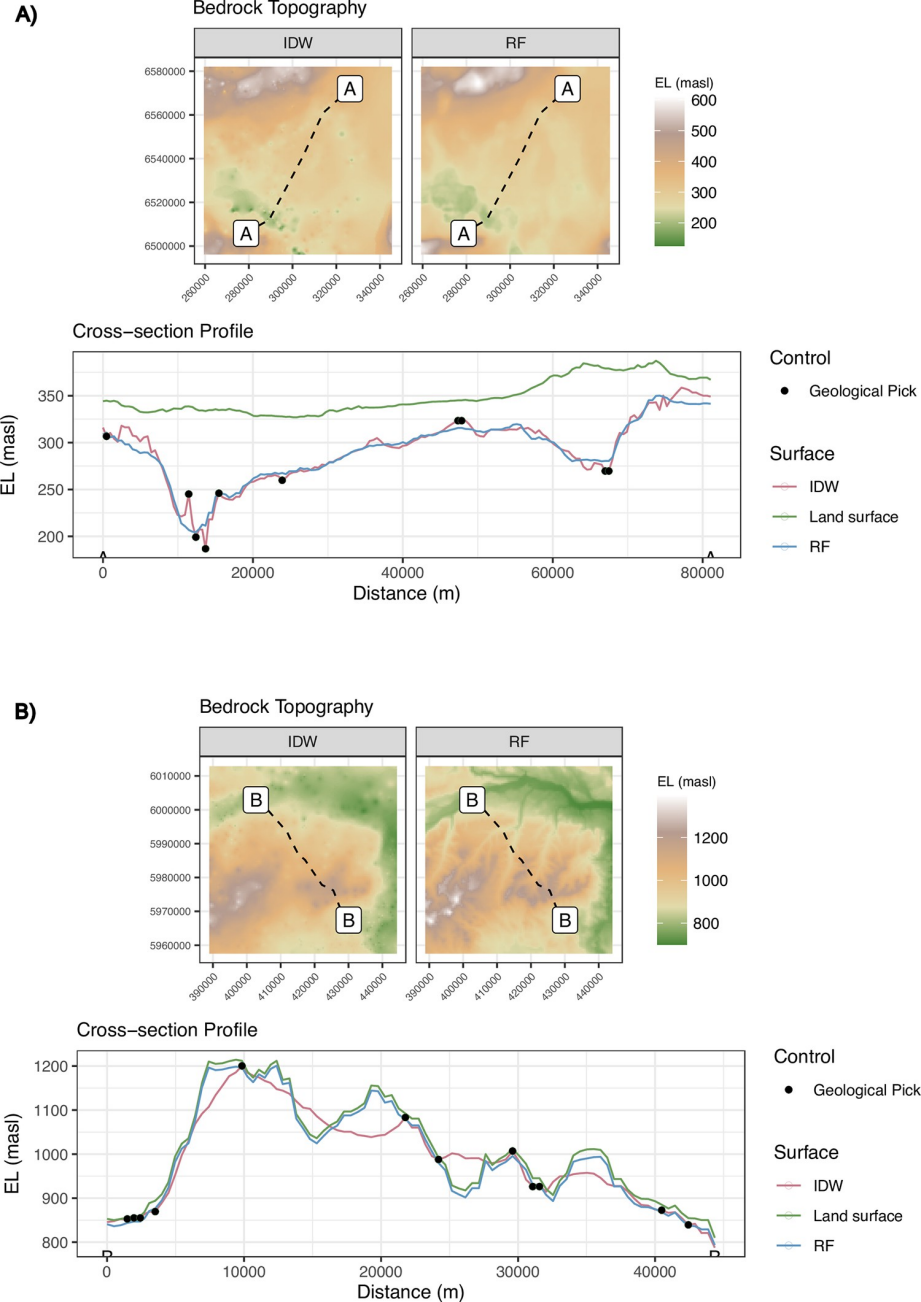
Comparison of the predicted DTB from the RF model with spatial interpolations of DTB using inverse-distance weighting interpolation (IDW). (a) Northern Alberta in lowland to moderate relief upland terrain. (b) West-central Alberta in upland to mountainous terrain.

Finally, the geomorphic form of the derived bedrock elevation surface after subtracting the machine learning predictions from the DEM was compared to the baseline methods when bedrock topography was interpolated directly (IDW-BSE) using maps and cross-sectional profiles through the WCAB and NWAB the sub-regions (Fig 13). In the NWAB sub-region, the form of the derived bedrock surface from the RF model is similar to that obtained from IDW interpolation, apart from it does not suffer from the ‘bullseye’ type artifacts that are visible in the IDW predictions. However, the overall similarity between the derived bedrock surface and IDW, even when the bedrock surface is deeply buried and shows little correlation with the surface topography, strongly supports that the machine learning approach can be used as a surrogate for long-established spatial interpolation methods for DTB and bedrock topography mapping.

In contrast, a comparison of cross-sectional profiles through the upland WCAB sub-region shows that although the geological setting is characterized by low DTB, and the bedrock topography mirrors the land surface, the IDW-BSE with no terrain information smoothly interpolates across major topographic features where data are sparse. This causes the interpolated bedrock surface to exceed the land surface elevation, and more problematically, the bedrock topography smoothly interpolates through high-amplitude variations in the terrain, generating erroneously large DTB estimates if the bedrock elevation is subtracted from the DEM. Predictions from the RF model in contrast closely follow the land surface topography.

## Discussion and conclusions

The predictive performances of several machine learning approaches for DTB mapping were systematically examined using topographic and land-surface spectral data in conjunction with several spatial feature engineering approaches to account for spatial dependence in the training data locations. We show that adding spatial lag variables provides clear benefits in terms of increased predictive performances for DTB mapping, while yielding maps that are geologically plausible and generally free of spurious spatial artifacts. This is particularly the case for regions that host thick sediments above bedrock, such as in the NWAB and SAOS sub-datasets. In these settings, the correlation between surface topography and bedrock topography is low, and therefore information from terrain and/or remote sensing predictors will be less relevant to predicting the topography of a deeply buried surface. The feature importance scores demonstrate this, with relatively low importance being assigned to these predictors, and instead, the models strongly leverage the spatial lag features. However, in upland and mountainous regions where moderate to strong correlation between surface and bedrock topography is expected (and is supported by high feature importances) the spatial lag feature engineering approach does not impede model performance, and the RMSE scores were still slightly reduced. Consequently, the proposed machine learning and spatial feature engineering approach is expected to be particularly valuable for mapping DTB across other regions that host thick overlying sediments, while being robust enough to be applied across any type of terrain.

This study also demonstrated that although some proposed methods of adding additional spatial variables to machine learning models does increase predictive performances, such as when using the T+EDF spatial feature engineering method, or when using neighbouring spatial points directly as features (T+NN), these methods can result in spatial artifacts in the predicted maps. For the T+NN approach, this is most likely caused by the model depending on the single closest neighbouring point, which is essentially a Thiessen polygon interpolation. The EDFs approach can also lead to ‘step-like’ patterns in the predicted maps because the use of linear distance measures does not guarantee a smooth prediction surface when using tree-based models. Similar issues have been noted in other studies when using buffer distances as features [50]. Furthermore, omitting information from neighbouring spatial locations also resulted in some spurious patterns, such as ‘inverted’ river channels, occurring in the predicted maps. These patterns were eliminated when spatial variables were included in the model. We interpret this to reflect the tendency of the machine learning models to strongly leverage the terrain features to adequately describe the spatial structure of the training data, even when these features are not providing useful information in deep sediment areas, which makes the models overly sensitive to spatial patterns in the raster grids.

In comparison to other approaches for DTB mapping, traditionally used spatial interpolation methods such as IDW and ordinary kriging suffer from the issue of needing to choose between interpolating DTB or bedrock surface elevation. The former approach is more suitable in mountainous and upland terrain where bedrock elevation is typically highly correlated with land surface elevation, while the latter is preferred in regions where the bedrock topography is uncorrelated from land surface topography, typical of many lowland regions. This is problematic in regions of mixed physiography, and most studies tend to either generate multiple separate models [9, 13, 14] or resort to an iterative process where the initial results are repeatedly re-interpolated with synthetic data that has been generated to resolve problematic areas [14, 51]. In either case, this makes the modelling process cumbersome and impossible to automate. In contrast, the proposed machine learning approach is fully automated, requiring no human intervention for model fitting, nor does it require subjective subdivisions to be made in the spatial data. This represents a major practical advantage of the proposed machine learning approach over other methods for large scale mapping because it can use information from a wide range of auxiliary variables in an automated manner, while also behaving similarly to other spatial interpolation methods in the extreme case of where the auxiliary predictors provide limited information for predicting DTB. Other advantages are the absence of requirements for spatial stationarity, and that the proposed approach can handle massive datasets and could be extended to global data through parallel and distributed computing.

Finally, there are several methodological aspects that may further improve predictive performances and warrant further studies, particularly in terms of using differing number of neighbours, additional combinations of spatial weight functions. Spatial lag features could also potentially be generated from the predictor variables rather than just aggregating information from the response variable (DTB) at surrounding spatial locations. This latter approach could help the model ‘learn’ new information from the predictors themselves, for example, using the variation in surface topography from neighbouring locations to assist in the prediction. The same approach has strong potential for any spatial classification models where the predicted categories exhibit a degree of spatial dependence on the location of training samples.

Overall, this study adds to growing body of research that the use of machine learning models with spatial features that account for spatial structure/autocorrelation provides an alternative and robust approach for making spatial predictions, and which in some cases are more practical that conventional spatial interpolation techniques. These methods do not aim to replace traditional spatial interpolation methods and there is no single ‘best’ approach because the results depend on the characteristics of each specific dataset. However, this study demonstrates that the proposed method represents an additional tool that is a strong candidate for mapping DTB across varied geological and physiographic settings and potentially could be extended to other types of spatial data.
